# Hypercalcemia in Small Lymphocytic Lymphoma with an Elevated Parathyroid Hormone-Related Peptide Associated with Early Richter Transformation

**DOI:** 10.1155/2021/5525721

**Published:** 2021-04-28

**Authors:** Ashish Bains, Pedro Covas, Olga Timofeeva, Michael Bromberg

**Affiliations:** ^1^Department of Pathology and Laboratory Medicine, Lewis Katz School of Medicine at Temple University, Philadelphia, PA, USA; ^2^Department of Internal Medicine, George Washington University, Washington, DC, USA; ^3^Department of Pathology, Georgetown University Hospital, Washington, DC, USA; ^4^Department of Internal Medicine, Section of Hematology, Lewis Katz School of Medicine at Temple University, Philadelphia, PA, USA

## Abstract

Hypercalcemia in malignancy is associated with multiple mechanisms and occurs in up to 20–30% of cancer patients. We report a case of small lymphocytic lymphoma/chronic lymphocytic leukemia (SLL/CLL) associated with hypercalcemia and an elevation in parathyroid hormone-related peptide (PTHrP) in the setting of a Richter transformation. Real-time reverse transcriptase PCR on lymph node biopsy specimens obtained before and after transformation showed an 8-fold increase in PTHrP mRNA levels and about 2-fold decrease in the levels of its cognate receptor PTHR1. The findings of this case suggest that parathyroid hormone-related peptide might be useful in monitoring a specific group of patients with SLL/CLL who develop hypercalcemia during the course of their disease and could suggest an autocrine-like mechanism involving PTHrP in Richter transformation.

## 1. Introduction

Hypercalcemia occurs in up to 20–30% of patients with malignancy [1]. One of the main contributors of hypercalcemia in malignancy is an elevation in parathyroid hormone-related peptide (PTHrP), most commonly seen in squamous cell carcinomas and breast and bladder cancers [1]. Small lymphocytic lymphoma/chronic lymphocytic leukemia (SLL/CLL) is a low-grade, indolent malignancy that usually is not associated with hypercalcemia or an elevated PTHrP. However, SLL/CLL can transform to an aggressive lymphoma, known as a Richter transformation (RT), which occurs about 5% of the time and usually carries a poor prognosis [2, 3]. We investigated the potential association of PTHrP in Richter transformation in a case involving a 70-year-old woman with SLL/CLL who developed hypercalcemia during the course of her disease.

## 2. Materials and Methods

An IRB approval was obtained to conduct testing on the available biopsy materials. The biopsy material used for RT-PCR was routinely processed, fixed in 10% formalin, and paraffin embedded. Total RNA was extracted from FFPE tissues (2 sections *x* 5 *μ*m thickness) using Qiagen FFPE RNeasy Kit and deparaffinization solution (Qiagen, Valencia, CA, USA) according to the manual. cDNA synthesis and qRT-PCR were carried out using the Invitrogen SuperScript IV One-Step RT-PCR System (ThermoFisher Scientific, Waltham, MA, USA). PTHR1R and PTHrP levels were quantified using TaqMan assays Hs00896826_g1 and Hs00174969_m1, respectively. The expression levels were normalized using GAPDH expression levels (Hs02786624) (ThermoFisher Scientific, Waltham, MA, USA). The comparative cycle threshold (CT) method was used to analyze the data by generating relative values of the amount of target cDNA (Applied Biosystems) using the following formula: 2−ΔCT, where CT represents the number of cycles for the amplification of target to reach a fixed threshold and correlates with the amount of starting material present; ΔCT = difference between the threshold cycles of the target and an endogenous reference (GAPDH).

## 3. Results

**Figure 1:**
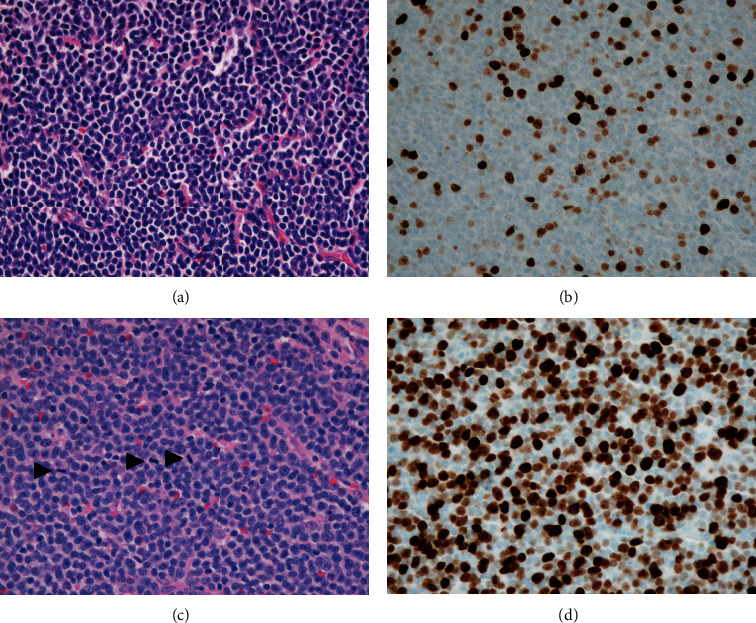
H&E sections of the lymph node excisional biopsy before transformation, demonstrating small round B-lymphocytes with clumped chromatin and rare mitosis (1a); Ki67 showing a low proliferation index (1b). 1c and 1d demonstrate increased cell size with numerous mitotic figures (arrowheads) and a significantly increased proliferation index by Ki67, respectively, at the time of early transformation. All images are taken at 400X magnification.

**Figure 2:**
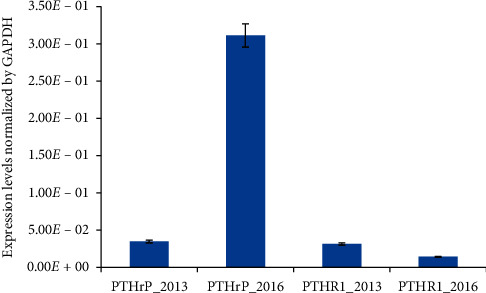
The mRNA expression of PTHrP and its receptor PTHR1 in the lymph nodes. The PTHrP levels increased, while PTHR1 levels have decreased in 2016 compared to 2013. The mRNA was extracted from formalin-fixed paraffin-embedded lymph node tissue collected in 2013 and 2016. The expression levels of PTHrT and PTHR1 were normalized using GAPDH expression levels. Each bar represents a mean of three independent experiments for one cell line; bars represent SD.

The patient initially presented in 2010 for evaluation of a left cervical mass in the absence of constitutional symptoms. An excisional biopsy of the left cervical lymph node revealed SLL without evidence of transformation. A metabolic panel at the time of diagnosis was normal and showed a total calcium of 9.2 mg/dL (normal 8.5–10.2 mg/dL), albumin of 4.4 gm/dL (normal 3.5–5.0 gm/dL), BUN 16 mg/dL (normal 8–20 mg/dL), and creatinine 0.94 mg/dL (normal 0.40–1.30 mg/dL). She was observed expectantly and did well until 2013, when the neck mass enlarged. A repeat biopsy of the mass showed SLL with a low proliferation rate (Ki67 approximately 10%) still without evidence of transformation (Figures 1(a) and 1(b)). Her calcium levels at that time and until late 2015 were normal (10.6 mg/dL). She was treated with rituximab and bendamustine for a total of four cycles, which yielded a partial response, and was closely observed until 2016 when she presented with dyspnea and generalized lymphadenopathy. CT scan evaluation revealed diffuse lymphadenopathy above and below the diaphragm. Her total and ionized calcium levels at this time and subsequently were elevated, 12.1 mg/dL and 5.6 mg/dL (normal 4.6–5.3 mg/dL), respectively. She was treated with aggressive intravenous fluid repletion and started on bisphosphonate therapy. PTH and PTHrP levels were 4 pg/mL (normal 10–65 pg/mL) and 151 pmol/L (normal <2 pmol/L), respectively. A follow-up excisional biopsy of a right axillary lymph node demonstrated persistent involvement by CLL/SLL with larger sized neoplastic B-cells, frequent mitotic activity, and a markedly increased proliferation index compared with the prior biopsies, with evidence of early RT (Figures 1(c) and 1(d)). At this time, her peripheral blood was involved by clonal lambda restricted B-cells with an absolute lymphocyte count of 1.6 K/uL and CD45+, CD19+, CD20+ dim, CD23+, and CD5+ immunophenotype by flow cytometry. Peripheral blood cytogenetic analysis demonstrated trisomy 8 and 13q deletion. Immunohistochemical stain on the biopsy material did not provide a definitive staining for PTHrP. However, qRT-PCR showed an 8-fold increase in PTHrP mRNA levels and about 2-fold decrease in the levels of its cognate receptor PTHR1 in 2016 compared with 2013 (Figure 2). These results suggest that the site of PTHrP production was in the lymph node and possibly involved the transformed lymphocytes.

The patient was treated with five cycles of R-CHOP chemotherapy and achieved a partial response. A follow-up PTHrP level was 46 pmol/L, at least a three-fold decline, with R-CHOP therapy. In 2017, she had a recurrence of her disease with persisting hypercalcemia (ionized calcium elevated up to 6.0 mg/dL) and at this time the total vitamin *D*, 1,25 (OH)_2,_ level was <17 pg/mL (normal range 18–72 pg/mL). She was treated with ibrutinib, but progressed, and electively chose hospice care.

## 4. Discussion

The etiology for transformation of an indolent small lymphocytic lymphoma into an aggressive variant is not fully known. There are possible risk factors including Epstein–Barr Virus infection and cytogenetic evolution such as trisomy 21 and chromosome 11 abnormalities. [2] Genetic markers such as polymorphisms of CD38, BCL2, and lipoprotein receptor-related protein 4 have also been shown as risk factors to developing a RT. *NOTCH1* mutation has also been noted to increase the risk for a RT. [4, 5].

The symptoms of RT are frequently nonspecific and there is no well-established biomarker to help with diagnosis. FDG/PET imaging can be of ancillary benefit in suspected RT, but FDG avid lymphadenopathy is also nonspecific and ultimately an excisional biopsy is required. [6, 7] Although hypercalcemia is not a common characteristic, it has been regarded in the literature as a precursor to RT. In a review of four case reports of patients with low-grade non-Hodgkin lymphomas, one of the two with CLL/SLL had an elevated PTHrP level nearly two times the upper limit of normal, possibly indicating an association.[8]. Another study described a patient with CLL/SLL with hypercalcemia and 2,500-fold higher peripheral blood PTHrP mRNA level compared to healthy controls at the time of RT.[9]. Other previous studies have also suggested a poorer outcome following the detection of hypercalcemia with higher incidence of histologic transformation and leukemic cell production of PTHrP that may have a pathogenic role in hypercalcemia observed as a rare complication of CLL/SLL [10, 11]. Our data further suggest that the transformed lymphocytes in the lymph node can be the source of PTHrP production. Further studies are needed to establish the correlation between timing of RT and increased PTHrP mRNA levels to determine whether this may be a novel biomarker for early large cell transformation of CLL/SLL.

## 5. Conclusion

PTHrP might be a novel biomarker to follow in patients with SLL/CLL; although rarely upregulated, in patients who develop hypercalcemia on routinely tested chemistry panels, an increase in PTHrP levels could suggest early RT. This case illustrates that PTHrP can be useful in monitoring a specific group of patients with CLL/SLL who develop hypercalcemia during the course of their disease and might suggest an autocrine-like mechanism involving PTHrP in early large cell transformation.

## References

[1] Stewart A. (2005). Hypercalcemia associated with cancer. *New England Journal of Medicine*.

[2] Tsimberidou A. M., Keating M. J. (2005). Richter syndrome: biology, incidence, and therapeutic strategies. *Cancer*.

[3] Montgomery N., Mathews S. (2016). Transformation in low grade B-cell neoplasms. *Surgical Pathology Clinics*.

[4] Rossi D., Gaidano G. (2016). Richter syndrome: pathogenesis and management. *Seminars in Oncology*.

[5] Vitale C., Ferrajoli A. (2016). Richter syndrome in chronic lymphocytic leukemia. *Current Hematologic Malignancy Reports*.

[6] Falchi L., Keating M. (2014). Correlation between FDG/PET, histology, characteristic, and survival in 332 patients with chronic lymphoid leukemia. *Blood*.

[7] Pemmaraju N., Jain P. (2017). PET-positive lymphadenopathy in CLL-Not always Richter Transformation. *Americal Journal of Hematology*.

[8] Beaudreuil J. (1997). Hypercalcemia may indicate Richter’s Syndrome. *Cancer*.

[9] Watanabe N. (2017). Richter’s syndrome with hypercalcemia induced by tumor- associated production of parathyroid hormone-related peptide. *Case Reports in Oncology*.

[10] Seymour J. F., Khouri I. F., Champlin R. E., Keating M. J. (1994). Refractory chronic lymphocytic leukemia complicated by hypercalcemia treated with allogeneic bone marrow transplantation. Case report and review. *American Journal of Clinical Oncology*.

[11] Seymour J. F., Keating M. J. (1995). Parathyroid hormone related protein in hypercalcaemia in CLL. *British Journal of Haematology*.

